# Acetylsalicylic Acid at Trace Concentrations Induces Cellular Toxicity and Phytotoxicity in the Roots of Cultivated Plants

**DOI:** 10.3390/toxics14090809

**Published:** 2026-09-11

**Authors:** Carla Rafaela Somera, Edson Araujo de Almeida, Matheus Cristiano Hermann Massariol, Diego Espirito Santo, Danielle Cristina da Silva de Oliveira, Adriele Rodrigues dos Santos, Gideã Taques Tractz, Regiane da Silva Gonzalez, Osvaldo Valarini, C. A. Downs, Ana Paula Peron

**Affiliations:** 1 Chemical Engineering Course, Federal Technological University of Paraná, Campo Mourão 87301-899, Paraná, Brazil; carlasomera@alunos.utfpr.edu.br (C.R.S.); massariol@alunos.utfpr.edu.br (M.C.H.M.); 2 Postgraduate Program in Chemistry, Maringá State University, Maringá 87020-900, Paraná, Brazil; pg55523@uem.br; 3 Postgraduate Program in Biological Sciences, State University of Londrina, Londrina 86057-970, Paraná, Brazil; diegoespst.1997@uel.br; 4 Academic Department of Chemistry, Federal Technological University of Paraná, Campo Mourão 87301-899, Paraná, Brazil; daniellesilva@utfpr.edu.br; 5 Experimental Coordination, Federal Technological University of Paraná, Campo Mourão 87301-899, Paraná, Brazil; adrielesantos@utfpr.edu.br; 6 Postgraduate National Network in Management and Regulation of Water Resources, Federal Technological University of Paraná, Campo Mourão 87301-899, Paraná, Brazil; gideatractz@utfpr.edu.br; 7 Postgraduate Program in Food Technology, Federal Technological University of Paraná, Campo Mourão 87301-899, Paraná, Brazil; regiane@utfpr.edu.br (R.d.S.G.); osvaldovalarini@utfpr.edu.br (O.V.J.); 8 Postgraduate Program in Technological Innovations, Federal Technological University of Paraná, Campo Mourão 87301-899, Paraná, Brazil; 9 Haereticus Environmental Laboratory, Gladstone, VA 24553, USA; cadowns@haereticus-lab.org

**Keywords:** emerging contaminants, environmentally relevant levels, oxidative stress, phytotoxicity, cytogenotoxicity, root development

## Abstract

Acetylsalicylic acid (ASA) is frequently detected in wastewater and sewage sludge, which may represent potential pathways of entry into agricultural environments. However, its effects on cultivated plants at trace concentrations remain unexplored. This study evaluated the effects of ASA (1–1000 ng·L^−1^) on germination and root growth of *Daucus carota*, *Solanum lycopersicum*, and *Cucumis sativus*, as well as cytotoxic and genotoxic effects in the root meristems of *Allium cepa* bulbs and biochemical responses associated with redox homeostasis in the roots of all four species. No significant adverse effects were observed at the lowest concentrations tested; however, exposure to 100 and 1000 ng·L^−1^ reduced root growth in all species, with a relative growth index below 0.8. In *A. cepa*, these concentrations induced mitodepressive effects, with mitotic indices below 70% relative to controls, and increased the frequency of chromosomal abnormalities, with total CAI values of 9.6% and 11.9%, respectively. At 100 ng·L^−1^, C-metaphases and chromosomal disorganization during prophase were observed, characterizing an aneugenic effect, whereas at 1000 ng·L^−1^, these abnormalities were accompanied by metaphases with sticky chromosomes, indicating a clastogenic effect. ASA also altered redox homeostasis in the roots of all four species, as indicated by concentration-dependent modulation of antioxidant enzymes, increased lipid peroxidation, and the absence of a compensatory non-enzymatic antioxidant response. These findings indicate that trace concentrations of ASA can impair early plant development and induce cytotoxic, genotoxic, and biochemical alterations in the roots of the species studied under the experimental conditions used. These findings emphasize the importance of assessing ASA effects under soil-based and field-relevant conditions to better understand its environmental implications.

## 1. Introduction

Acetylsalicylic acid (ASA), chemically known as 2-acetoxybenzoic acid, is a synthetic drug widely used as an analgesic, antipyretic, anti-inflammatory, and antiplatelet agent. Its low production cost, combined with high therapeutic efficacy, has driven its use since the late 19th century, establishing it as one of the most widely consumed medications worldwide [1,2]. Currently, this compound is included in the World Health Organization’s [3] list of essential medicines, with an international market valued at USD 2.8 billion in 2025 and projections to exceed USD 3 billion in the coming decade [4], reflecting its continued consumption and its consequent entry into environmental compartments, particularly aquatic and edaphic ones.

Classified as a micropollutant, ASA has been detected in wastewater at concentrations ranging from sub-µg·L^−1^ to the µg·L^−1^ range, with reported values reaching up to 220 µg·L^−1^ [5,6,7]. In sewage sludge, ASA has been reported at concentrations ranging from 0.374 to 367.0 mg·kg^−1^ [8]. It is noteworthy that the annual global production of domestic sewage sludge is estimated at approximately 45 million tons of dry matter, of which more than half is applied to agricultural soils for fertilization purposes [9,10]. Furthermore, the use of wastewater for agricultural irrigation is widespread, especially in water-scarce regions, and represents a continuous pathway for the introduction of micropollutants into agricultural soils [11,12,13]. These practices may therefore contribute to the recurrent introduction of ASA into agricultural environments, although information on its occurrence in agricultural soils remains limited.

The environmental behavior of ASA is influenced by its ionizable acidic nature and susceptibility to transformation [14]. ASA has a relatively low octanol–water partition coefficient (log Kow ≈ 1.2) and a pKa of approximately 3.5, properties that influence its ionization, sorption, and distribution among environmental compartments [15]. Available evidence indicates that ASA is susceptible to abiotic and biological transformation, with reported degradation rates and half-lives varying according to environmental conditions [16]. Although ASA is not considered highly persistent, its continuous input into agricultural environments may result in recurrent exposure. However, its degradation kinetics and environmental fate in agricultural soils remain poorly characterized.

Studies on plant exposure to ASA have primarily focused on high concentrations (µM to mM), in which the compound is used as a physiological modulator associated with the induction of tolerance to abiotic stresses, such as salinity, drought, and oxidative stress [17,18]. In contrast, the ecotoxicological effects of ASA at environmentally relevant residual concentrations remain largely unexplored. This knowledge gap is particularly relevant to the soil aqueous phase, which represents a highly dilute environmental compartment where freely dissolved contaminants generally occur at trace levels, often in the ng·L^−1^ range [13]. Although direct measurements of ASA in agricultural soil solutions remain scarce, the presence of contaminants at such trace concentrations in this compartment provides an appropriate environmental context for investigating the effects of residual pharmaceuticals on plants. Moreover, generating ecotoxicological data at trace concentrations is essential for reducing uncertainties in the environmental assessment of pharmaceuticals in agricultural systems and for supporting future regulatory strategies [19,20,21].

Oxidative stress has been proposed as an important mechanism involved in plant responses to ASA exposure. Previous studies have shown that, at relatively high micromolar to millimolar concentrations, ASA modulates antioxidant metabolism, including enzymatic responses involving dismutases and peroxidases, as part of the cellular response to abiotic stress [17,18]. However, no studies have investigated whether residual concentrations of ASA can cause biochemical alterations indicative of oxidative stress in plants. This represents an important knowledge gap because oxidative disturbances may contribute to phytotoxicity, while early sublethal biochemical alterations at trace concentrations may impair root development and compromise subsequent plant growth. Therefore, evaluating antioxidant responses and lipid peroxidation in plants exposed to trace concentrations of ASA is important for identifying early biochemical and cellular disturbances and improving the mechanistic understanding of its phytotoxicity.

*Daucus carota* L., *Solanum lycopersicum* L., and *Cucumis sativus* L. are widely used as bioassay models in ecotoxicological studies and are recommended by international guidelines, such as those of the United States Environmental Protection Agency [22] and the Organization for Economic Co-operation and Development [23], for assessing the adverse effects of environmental contaminants. These organisms allow investigation of changes in germination and meristematic tissues and are considered sensitive systems for detecting biological effects induced by contaminants, even at trace levels [24,25]. The roots of *Allium cepa* L., in turn, have been widely used in ecotoxicological studies due to their high sensitivity to chemical agents, allowing the detection of phytotoxic, cytotoxic, and genotoxic effects even at residual levels [26,27,28]. This model enables the integrated assessment of different levels of biological organization (morphological, physiological, cytogenetic, and biochemical), including responses related to oxidative stress, and shows high agreement with results obtained in other experimental systems [12,27,28,29,30,31,32].

Therefore, this study evaluated the effects of ASA, at concentrations potentially representative of the soil aqueous phase (ng·L^−1^), on germination, root growth, morphology, and antioxidant responses in *D. carota*, *S. lycopersicum*, and *C. sativus*. Additionally, in *A. cepa* roots, phytotoxic, cytotoxic, and genotoxic effects were investigated, together with antioxidant responses and lipid peroxidation, to further characterize the cellular effects of ASA at environmentally relevant concentrations. It was hypothesized that exposure of cultivated plants to trace concentrations of ASA would impair root development and cellular integrity and alter biochemical responses associated with redox homeostasis.

## 2. Materials and Methods

### 2.1. Obtaining the ASA, Defining and Preparing Exposure Concentrations

Acetylsalicylic acid (ASA; CAS 50-78-2; molecular weight 180.16 g·mol^−1^; log K_o_w ≈ 2.2–2.3) was obtained in analytical grade from Sigma-Aldrich, as were the other reagents used in this study.

The concentrations tested (1, 10, 100, and 1000 ng·L^−1^) were selected to represent an environmentally relevant range of ASA in the soil solution, simulating an exposure gradient at trace levels.

The concentrations tested were obtained by diluting a stock solution of ASA (0.03 g·L^−1^) prepared in the presence of Tween 80 (0.03 g·L^−1^), at pH 7.0 and 25 °C, with minimal exposure to light. Tween 80 was used as a vehicle for ASA to facilitate the preparation of the stock solution, given its solubilization capacity and relatively low toxicity, which support its widespread use as a non-ionic surfactant in environmental applications [33]. Thus, the concentration of Tween 80 in the treatment solutions decreased proportionally with the ASA concentration. A Tween 80 vehicle control (T80) was included at 1000 ng·L^−1^, corresponding to the highest Tween 80 concentration present in the ASA treatments. The T80 treatment was used to assess potential effects of the surfactant under the experimental conditions. No significant differences were observed between the T80 treatment and the distilled-water negative control.

### 2.2. Stability Analysis of ASA in Aqueous Media

Due to the susceptibility of ASA to hydrolysis in aqueous media and its potential conversion to salicylic acid, a stability analysis of the compound was conducted throughout the experimental period to verify the maintenance of exposure during the bioassays. The analytical procedures were adapted from the protocol described by [34].

First, a spectrophotometric analysis (using a UV-1800 UV–Vis spectrophotometer (Shimadzu Corporation, Kyoto, Japan) was performed on the 0.03 g·L^−1^ ASA standard solution (wavelength range 200–1000 nm) in comparison with salicylic acid. Based on the absorption curve, the maximum absorption wavelengths of the ASA compounds and salicylic acid were determined, as well as the possible hydrolysis of ASA.

The quantification of salicylic acid, resulting from the hydrolysis of ASA, was evaluated by spectrophotometric analysis of the salicylic acid-Fe(III) complex. To this end, 1.25 mL of a 2 × 10^−3^ mol·L^−1^ ASA solution was added to a test tube, followed by 1 mL of an 8 × 10^−3^ mol·L^−1^ Fe(III) solution. After mixing, the resulting solution was transferred to a quartz cuvette and analyzed by spectrophotometry over 200–1000 nm. The quantification of acetylsalicylic acid present in the ASA sample was performed using an analytical calibration curve (with concentrations ranging from 1.2 × 10^−3^ to 2 × 10^−4^ mol·L^−1^) obtained at 525 nm from a standard solution of salicylic acid (2 × 10^−3^ mol·L^−1^) in the presence of the Fe(III) solution.

The stability of ASA in aqueous solution was monitored over 7 days, corresponding to the exposure time used in the biological assays (Section 2.3.1 and Section 2.3.2), using the stock solution described above.

The method was validated using a standard [35] curve (Abs = 7.29.104.[ASA] + 0.00595, R = 0.999), in which the deviation and slope values of the line were used to determine the limits of detection (LOD) and quantification (LOQ) [36] of 2.7 × 10^−7^ ug·L^−1^ and 2.5 × 10^−6^ ug·L^−1^, respectively.

The change in concentration over time was determined using Equation (1), with the results expressed as a percentage of the initial concentration (time zero).

(1) $$\mathrm{Stability}\;(\%)=\frac{sample\;absorbance\;}{absorbance\;(day\;zero)\;}\times 100$$

### 2.3. Plant Tests

#### 2.3.1. Assessment of Phytotoxicity in Seeds of *D. carota*, *S. lycopersicum*, and *C. sativus*

Seeds of *D. carota*, *S. lycopersicum*, and *C. sativus* were obtained from commercial suppliers. The seeds were untreated, non-GMO, and had a minimum germination capacity of 95% and a purity of 99%, according to the information provided by the manufacturer.

Phytotoxicity was evaluated based on seed germination and root growth, according to the procedure described in [23]. For each treatment, 20 seeds were evenly arranged on filter paper placed in pre-sterilized Petri dishes. Five independent Petri dishes were used for each treatment, resulting in 100 seeds evaluated per treatment.

Before seed placement, the filter paper was uniformly moistened with 1.5 mL of the corresponding test solution. This volume was established to provide sufficient moisture for seed germination while avoiding excessive wetting of the substrate. After the seeds were distributed, the Petri dishes were sealed with plastic film and maintained in a BOD incubator (Biochemical Oxygen Demand incubator) at 25 °C under dark conditions for 7 days.

All procedures involving seed placement and handling of the experimental units were performed under low-light conditions to minimize exposure to ambient light during the assay. At the end of the incubation period, the germination percentage (G%) was determined for each treatment (Equation (2)), with seeds showing radicle protrusion considered to have germinated.

(2) $$G\left(\%\right):\frac{number\;of\;seeds\;germinated\;}{number\;of\;seeds\;evaluated\;}\times 100$$

After seven days of exposure, the roots were measured using a caliper. The measurements obtained for each treatment were then used to determine the Relative Growth Index (RGI) according to Equation (3).

(3) $$RGI=\frac{RLI}{RLC}$$

where RGI represents the Relative Growth Index, RLI corresponds to the mean root length measured in the exposed groups, and RLC represents the mean root length recorded for the control.

Based on the criteria described by [37], RGI values between 0.8 and 1.2 are considered indicative of no relevant effect on root growth. Values below 0.8 are associated with root elongation inhibition, whereas values above 1.2 indicate root growth stimulation.

The Germination Index (GI) was determined using Equation (4). Following the classification proposed by [38], GI values of ≤50% are classified as indicative of a lethal phytotoxic risk, values > 50% and <80% indicate phytotoxic risk, and values ≥ 80% are considered indicative of no phytotoxic risk.

(4) $$GI\left(\%\right)=\frac{RLI\times GSI\;}{RLC\times GSC\;}\times 100$$

In Equation (4), GI denotes the Germination Index. RLI refers to the mean root length recorded for the exposed seeds, whereas RLC represents the corresponding mean value obtained for the control group. GSI indicates the number of germinated seeds in each treatment, and GSC denotes the number of germinated seeds observed in the control.

Root morphology was also examined qualitatively based on changes in features including root shape, thickness, coloration, and mechanical consistency.

#### 2.3.2. Assessment of Phytotoxicity, Cytotoxicity, and Genotoxicity in *A. cepa* Roots

The effects of the tested compounds at the cellular and systemic levels were assessed in *A. cepa* roots following the methodology described by [39], with modifications based on the protocol of [40]. The bulbs were commercially obtained from an organic produce retailer. Prior to the assay, the dry outer scales were removed, and the bulbs were washed with distilled water to eliminate residual impurities from the surface.

For the exposure period, each bulb was placed individually in a beaker containing the corresponding test solution. The basal plate was immersed to ensure continuous contact with the solution and adequate conditions for root formation. The solutions were freshly prepared and replaced daily during the experiment. The bulbs were maintained for seven days in a BOD incubator at 25 °C in the absence of light. Five bulbs were used as independent experimental units for each treatment, and distilled water was used as the negative control.

At the end of the seven-day exposure, ten roots were randomly selected from each bulb and measured. Root length was measured with a caliper, and the Average Root Length (ARL) was calculated for each treatment using Equation (5).

(5) $$ARL\;(cm):\;\frac{Sum\;of\;root\;length\;of\;root\;bundles}{10}$$

Root morphology was additionally evaluated based on visual characteristics, including root form, diameter, pigmentation, and mechanical consistency.

For the cytotoxicity and genotoxicity evaluations, approximately five roots were collected from each bulb and immediately immersed in Carnoy’s fixative (3:1, *v*/*v*), where they remained for at least 12 h. Following fixation, the meristematic portions were excised and used for slide preparation. The resulting slides were examined by light microscopy at 400× magnification (Zeiss, Germany, São Paulo, SP, Brazil).

Cytotoxicity was assessed by scoring 10,000 cells for each treatment, corresponding to 2000 cells per bulb, and the Mitotic Index (MI) was obtained using Equation (6). Genotoxicity was evaluated from a total of 2000 cells per treatment, with 400 cells analyzed from each bulb. The Cellular Alteration Index (CAI) was subsequently determined according to Equation (7).

Chromosomal abnormalities indicative of genotoxicity were evaluated according to established cytogenetic criteria, including C-metaphases, chromosomal disorganization during prophase, anaphase and telophase bridges, sticky chromosomes during metaphase, and micronuclei [26]. The overall CAI was calculated for each treatment concentration. For treatments where a significant number of chromosomal abnormalities were observed, CAI values for each specific type of abnormality were additionally determined separately, using the same calculation method.

(6) $$\;\;\;MI:\;\frac{Total\;number\;of\;dividing\;cells}{Total\;number\;of\;cells\;analyzed}\times 100$$

(7) $$CAI:\;\frac{Number\;of\;cellular\;alterations}{2000}\times 100$$

### 2.4. Biochemical Analyses of D.carota, S. lycopersicum, C. sativus and A. cepa, Roots Exposed to Mixed Compounds

#### 2.4.1. Preparation of Enzymatic Extracts

For each experimental replicate, 50 mg of roots were excised as described in Section 2.3.2. The collected material was homogenized in 3 mL of potassium phosphate buffer (50 mM, pH 7.0) supplemented with 5 mM DPTA. The homogenates were then centrifuged at 4000 rpm for 15 min at 4 °C (Thermo Fisher Scientific, São Paulo, SP, Brazil) and the resulting supernatants were collected and used for the enzymatic assays.

Antioxidant enzyme activities were subsequently evaluated using these extracts. The enzymes analyzed were catalase (CAT), ascorbate peroxidase (APX), guaiacol peroxidase (GPOX), and superoxide dismutase (SOD).

#### 2.4.2. Enzymatic Assays

Catalase activity was determined following the procedure originally proposed by [41] and subsequently modified by [42]. Briefly, 100 µL of enzymatic extract from each replicate was mixed with 2.5 mL of sodium phosphate buffer (pH 7.8) and 1 mL of 1 mM H_2_O_2_. The decrease in absorbance associated with H_2_O_2_ decomposition was monitored at 240 nm using a UV–Vis spectrophotometer UV-1800 UV–Vis spectrophotometer (Shimadzu Corporation, Kyoto, Japan). CAT activity was calculated using a molar extinction coefficient of 2.8 M^−1^·cm^−1^ and expressed as µmol·min^−1^·µg^−1^ protein, according to Equation (8).

(8) $$\;U=\frac{\left[\frac{\left(\frac{A}{t}\right)}{E}\right]\times V_{e}\times DF}{P}$$

In Equation (8), U represents the enzymatic activity; A is the absorbance recorded during the assay; t corresponds to the reaction time; E is the molar extinction coefficient; Ve denotes the volume of enzymatic extract; DF is the dilution factor; and P represents the protein content calculated from the mass of root tissue used in the extraction.

APX activity was evaluated using the procedure reported by [43], with the modifications adopted in the present study. The reaction mixture consisted of 100 µL of enzymatic extract, 2.5 mL of sodium phosphate buffer, 500 µL of ascorbic acid (0.25 mM), and 1 mL of H_2_O_2_ (1 mM). Absorbance changes were monitored at 290 nm using a UV–Vis spectrophotometer. APX activity was calculated using a molar extinction coefficient of 2.8 M^−1^·cm^−1^ and expressed as µmol·min^−1^·µg^−1^ protein, according to Equation (8).

GPOX activity was assayed following the procedure described by [44]. The reaction mixture was prepared by combining 300 µL of enzymatic extract with 2.5 mL of sodium phosphate buffer, 250 µL of citric acid (0.1 M), 250 µL of guaiacol (0.5%), and 250 µL of H_2_O_2_ (1 mM). After mixing, the samples were maintained at 30 °C for 15 min and subsequently placed in an ice bath for 10 min. The reaction was terminated by adding 250 µL of sodium metabisulfite (2%). Absorbance was then recorded at 450 nm using a UV–Vis spectrophotometer. GPOX activity was calculated using a molar extinction coefficient of 26.6 M^−1^·cm^−1^ and expressed as µmol·min^−1^·µg^−1^ protein according to Equation (8).

SOD activity was measured based on the method reported by [45]. For each experimental replicate, the enzymatic extract was divided into two aliquots. One aliquot was illuminated with fluorescent light (80 W) for 20 min, whereas the second aliquot was maintained under dark conditions. For the enzymatic reaction, 200 µL of extract was added to a reaction mixture containing 0.8 mL of sodium phosphate buffer, 500 µL of EDTA (0.1 mM), 500 µL of methionine, 500 µL of nitroblue tetrazolium (NBT), and 200 µL of riboflavin. The absorbance was determined at 560 nm using a UV–Vis spectrophotometer. SOD activity was expressed as enzyme units per amount of protein, as calculated using Equation (9).

(9) $$SOD=\frac{\left(\frac{B_{l}-s_{l}}{B_{l}}\right)-\left(\frac{B_{e}-s_{e}}{B_{e}}\right)}{50}$$

In the calculation, Bl denotes the absorbance of the light-exposed blank prepared without enzymatic extract, whereas Sl corresponds to the absorbance of the light-exposed sample. Be represents the absorbance of the dark-incubated blank, and Se is the absorbance measured for the corresponding dark-incubated sample. The factor 50 refers to the amount of enzyme required to achieve 50% inhibition of NBT photoreduction.

#### 2.4.3. Non-Enzymatic Biochemical Assay

##### Sample Preparation

For the non-enzymatic antioxidant analyses, 50 mg of roots was collected from each experimental replicate, following the procedure described in Section 2.3.2. The tissue was suspended in 3 mL of distilled water and homogenized. The homogenates were subsequently centrifuged at 4000 rpm for 15 min, and the supernatant was collected for use in the antioxidant assays.

##### Antioxidant Activity Assay (DPPH)

The radical-scavenging capacity of the extracts was evaluated using the DPPH method described by [46]. Briefly, 50 µL of extract was combined with 250 µL of DPPH solution (0.00316% *w*/*v*). The reaction mixtures were maintained in the dark for 30 min at room temperature. Absorbance was then recorded at 515 nm using a UV–Vis spectrophotometer. The percentage of antioxidant activity was determined according to Equation (10).

(10) $$A\left(\%\right)=\frac{A_{c}-A_{s}}{A_{c}}\times 100$$

In Equation (10), AA% represents the antioxidant activity. The absorbance of the DPPH reagent alone is denoted by Ac, while As corresponds to the absorbance obtained for the reaction containing the sample and DPPH.

##### Determination of Phenolic Compound Content (Folin–Ciocalteu)

Total phenolic compounds were quantified following the procedure reported by [47]. For each replicate, 50 µL of homogenate was combined with 50 µL of distilled water and 50 µL of ethanol. The reaction was initiated by adding 100 µL of Folin–Ciocalteu reagent at 23 mM. The reagent was prepared by dissolving phosphotungstic acid and phosphomolybdic acid in methanol.

The reaction mixtures were initially incubated at room temperature in the absence of light for 10 min. A 50 µL aliquot of saturated sodium bicarbonate was then added to each mixture, followed by a second incubation in the dark for 50 min. Absorbance was subsequently recorded at 745 nm using a UV–Vis spectrophotometer. Total phenolic content was quantified using gallic acid as the reference standard.

##### Lipid Peroxidation Assay (TBARS)

Lipid peroxidation was determined using the method described by [48]. For the analysis, 50 µL of the homogenate from each replicate was added to 250 µL of TBARS solution (46 mM). The samples were incubated in a water bath at 90 °C for 35 min, then cooled to room temperature. Following this procedure, absorbance was determined by UV–Vis spectrophotometry at 532 nm, and lipid peroxidation was expressed in mol using malondialdehyde (MDA) as the analytical standard.

### 2.5. Statistical Analysis

Statistical analyses of the phytotoxicity, cytotoxicity, genotoxicity, and biochemical data were performed using the Kruskal–Wallis nonparametric test, followed by Dunn’s test for multiple comparisons. Statistical significance was set at *p* ≤ 0.05. The nonparametric approach was selected because the data did not meet the normality assumption, as assessed by the Lilliefors test. Analyses were performed in RStudio using the R statistical environment (RStudio version 2023.12.1; Posit Software, PBC, Boston, MA, USA) and the R statistical environment (version 4.3.2) [49].

## 3. Results and Discussion

### 3.1. Stability of ASA in Aqueous Media

**Figure 1:**
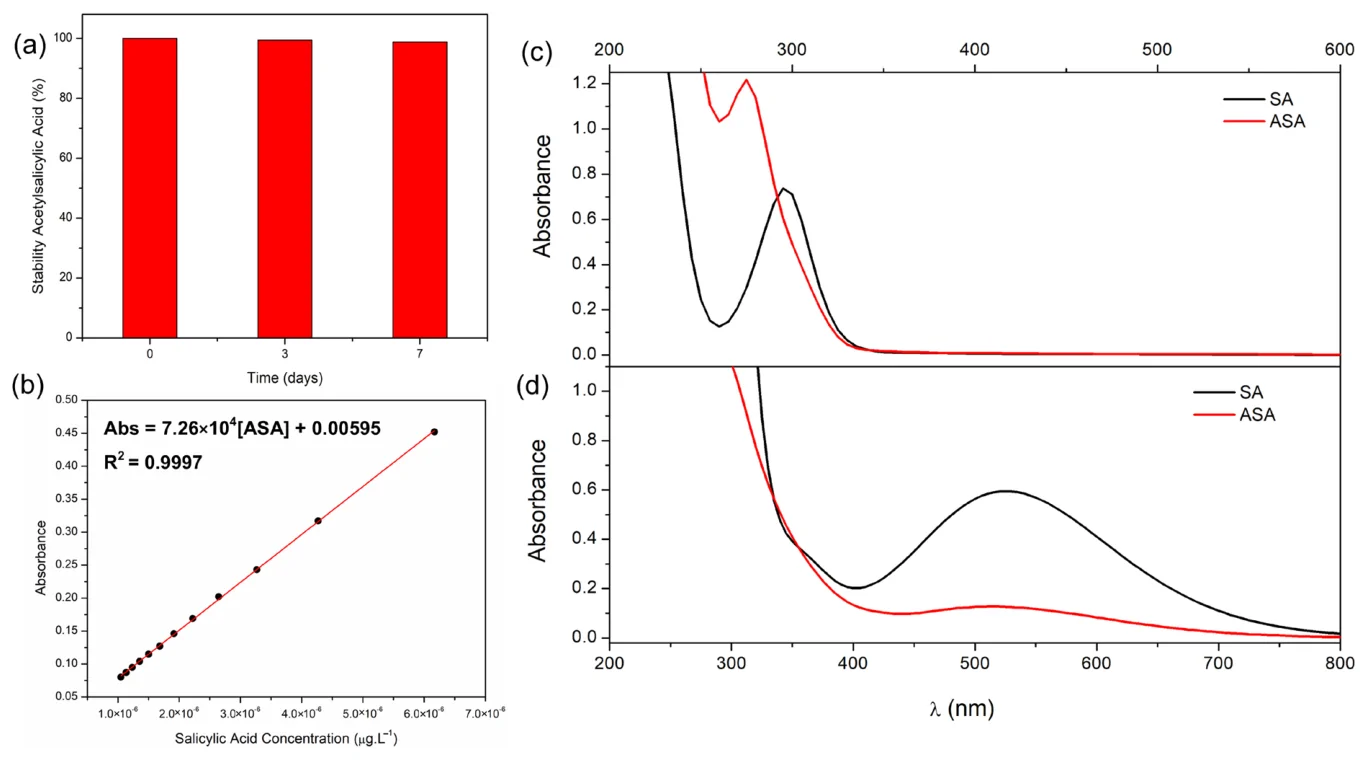
(**a**) Stability of acetylsalicylic acid in aqueous solution over a period of seven days; (**b**) analytical curve of the salicylic acid–Fe(III) complex; (**c**) UV–Vis absorption spectra of SA and ASA; (**d**) spectra of the salicylic acid–Fe(III) complex obtained for SA and ASA. ASA—acetylsalicylic acid. SA—salicylic acid.

Given the potential for ASA to undergo hydrolysis and convert to salicylic acid, a spectrophotometric analysis of the compound was initially performed over 200–1000 nm. The absorption spectrum of ASA did not show characteristic bands of salicylic acid, as evidenced by the comparison of the spectral profiles of the two compounds (Figure 1c).

However, trace amounts of salicylic acid in solution may not be detected solely by direct spectral scanning, necessitating the use of more sensitive analytical methods. Figure 1d shows the UV–Vis absorption spectrum of the salicylic acid–Fe(III) complex. When salicylic acid interacts with Fe(III) ions, a maximum absorption band at 525 nm is observed, characteristic of complex formation. This signal was also detected in the ASA solution; however, with low intensity, indicating minimal formation of salicylic acid resulting from the hydrolysis of ASA.

To quantify the salicylic acid content formed by hydrolysis, an analytical calibration curve for the salicylic acid–Fe(III) complex was constructed over the concentration range used in the study (Figure 1b). Using the equation of the straight line, the concentration of salicylic acid in the ASA solution could be determined. After seven days, the solution contained less than 1% salicylic acid derived from hydrolysis, indicating high stability of the ASA under the experimental conditions adopted. These results demonstrate that, throughout the entire experimental period, including the biological, biochemical, and stability assays, the purity of the ASA remained above 99%.

### 3.2. Phytotoxic Potential in Seeds and Phytotoxic, Cytotoxic, and Genotoxic Potential in the Roots of A. cepa Bulbs

**Figure 2:**
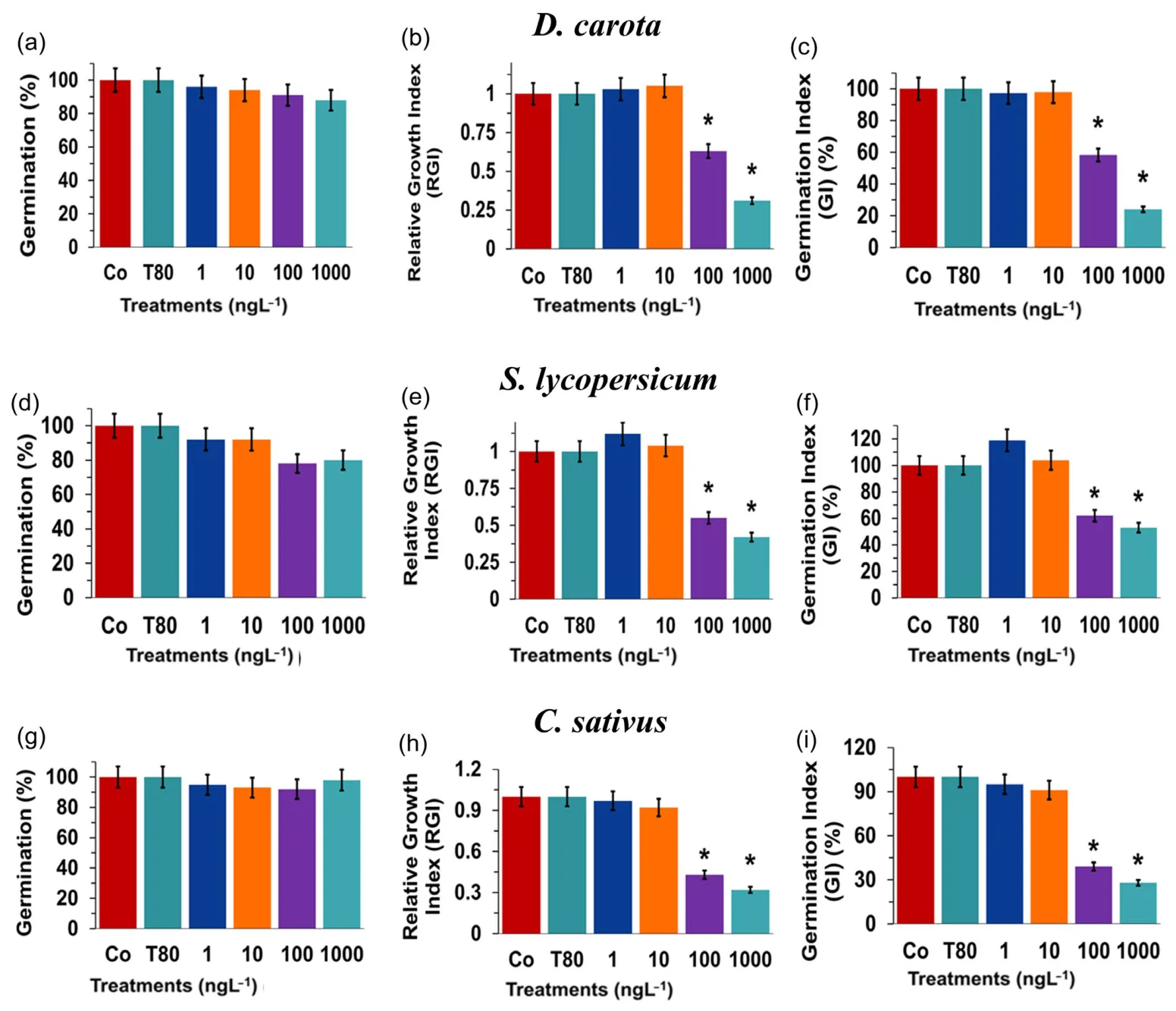
Phytotoxicity of acetylsalicylic acid on seeds of *Daucus carota* L., *Solanum lycopersicum* L., and *Cucumis sativus* L. at concentrations of 1, 10, 100, and 1000 ng·L^−1^, based on the parameters of seed germination and Relative Growth Index. * Significant differences from the distilled water control according to Kruskal–Wallis H, followed by Dunn’s post hoc test (*p* ≤ 0.05). Co—Distilled water control. T80—Tween 80 at a concentration of 1000 ng·L^−1^. (**a**) Germination in *D. carota*; (**b**) Relative Growth Index in *D. carota*; (**c**) Germination Index in *D. carota*; (**d**) Germination in *L. esculentum*; (**e**) Relative Growth Index in *L. esculentum*; (**f**) Germination Index in *L. esculentum*; (**g**) Germination in *C. sativus*; (**h**) Relative Growth Index in *C. sativus*; (**i**) Germination Index in *C. sativus*.

ASA, at concentrations of 1 and 10 ng·L^−1^, did not affect germination or root elongation in *D. carota*, *S. lycopersicum*, and *C. sativus* (Figure 2a,d,g), with GI values equal to or higher than those of the control (Figure 2c,f,i). In contrast, at concentrations of 100 and 1000 ng·L^−1^, although germination was not significantly altered, root growth was significantly reduced (Figure 2b,e,h), resulting in GI values of 24.05–79.05%, indicating phytotoxic potential.

**Figure 3:**
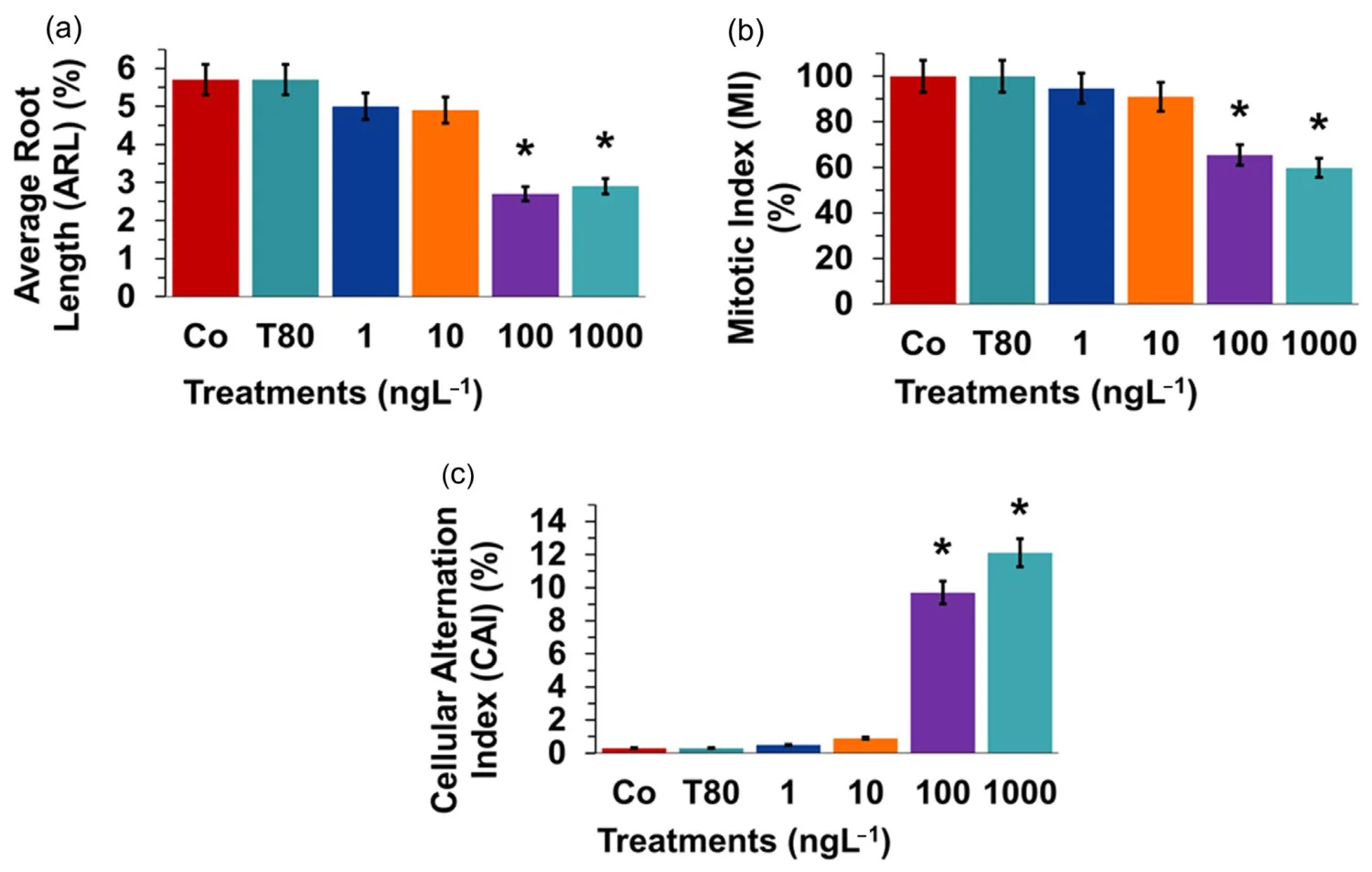
Phytotoxicity, cytotoxicity, and genotoxicity of acetylsalicylic acid in *Allium cepa* L. roots at concentrations of 1, 10, 100, and 1000 ng·L^−1^, based on the parameters Average Root Length (ARL), Mitotic Index (MI), and Cellular Alteration Index (CAI). * Significant difference from the distilled water control according to Kruskal–Wallis H, followed by Dunn’s post hoc test (*p* ≤ 0.05). Co—Distilled water control. T80—Tween 80 at a concentration of 1000 ng·L^−1^. (**a**) Average Root Length (ARL); (**b**) Mitotic Index (MI); (**c**) Cellular Alterations Index.

Under the conditions evaluated, germination maintenance shows lower sensitivity to this process than root growth, possibly due to the greater structural and functional protection of the embryonic axis during the early stages of plant development [50,51]. On the other hand, root development tends to be more susceptible to the action of xenobiotics, since the elongation zone exhibits high metabolic activity and greater exposure to the external environment, favoring interaction with exogenous compounds [50,51,52]. This pattern in radicle elongation in *D. carota*, *S. lycopersicum*, and *C. sativus* (Figure 2) was corroborated by results in *A. cepa* bulbs, in which ASA, at concentrations of 100 and 1000 ng·L^−1^, also promoted a significant reduction in root growth (Figure 3a).

In *A. cepa*, ASA at concentrations of 1 and 10 ng·L^−1^ did not cause cellular toxicity in the roots (Figure 3b,c). However, at concentrations of 100 and 1000 ng·L^−1^, it induced a significant mitotic-depressing effect in the root meristems, demonstrating cytotoxic potential (Figure 3b). The significant reduction in the mitotic index indicates interference in cell cycle progression, possibly due to disturbances in DNA replication and/or in the regulation of checkpoints between interphase phases, compromising the proliferative activity of meristematic cells and, consequently, root growth [26,29,53].

**Figure 4:**
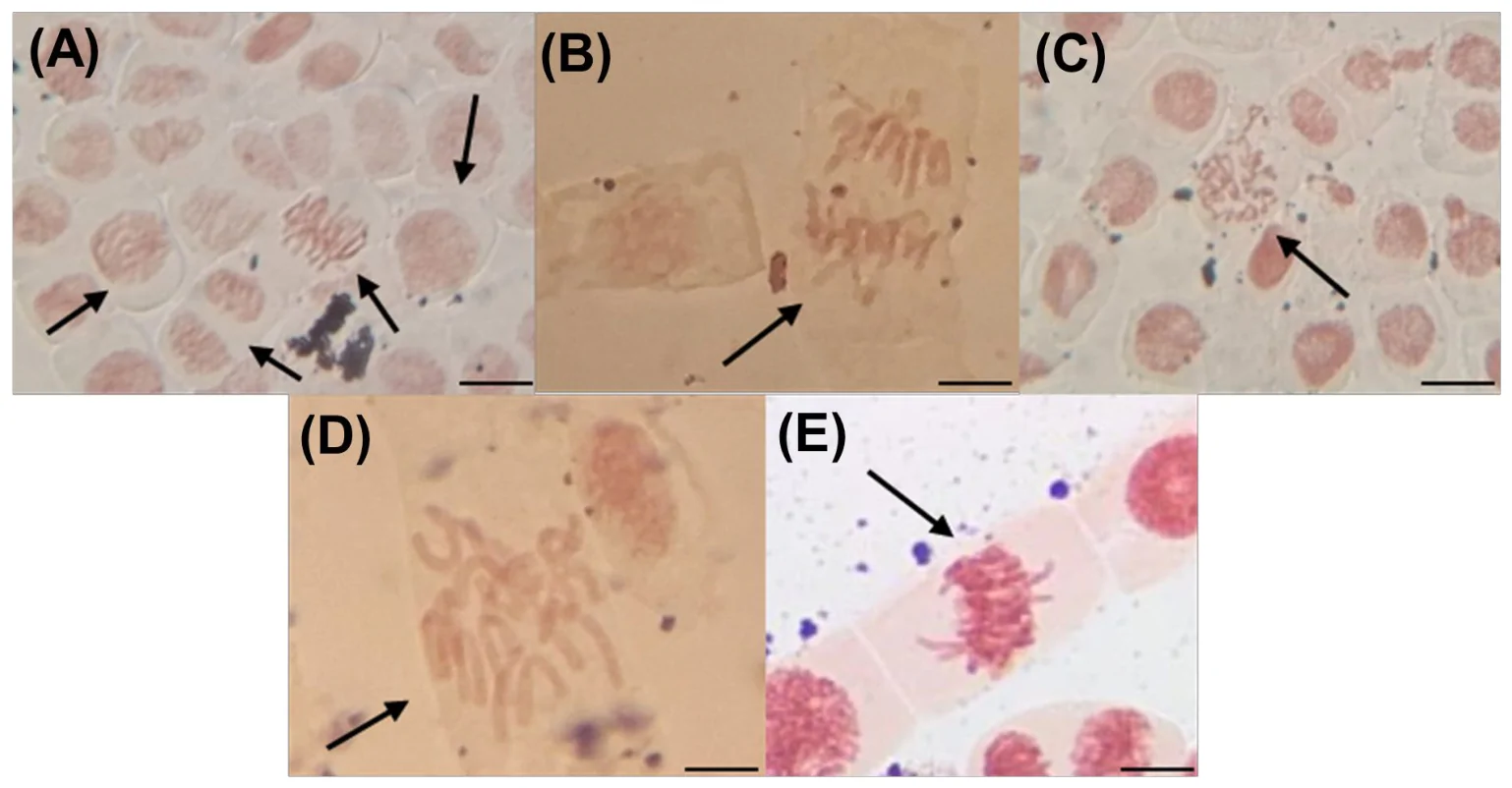
Cellular changes observed in root meristems of *Allium cepa* L. bulbs treated with acetylsalicylic acid at concentrations of 1, 10, 100, and 1000 ng·L ^−1^: (**A**) Normal cells showing, from left to right, prophase, telophase, metaphase and interphase, indicated by arrows; (**B**) normal anaphase; (**C**) chromosomal disorganization in prophase; (**D**) C-metaphase; and (**E**) sticky chromosomes at metaphase. Scale bar: 10 µm.

In addition, ASA at concentrations of 100 and 1000 ng·L^−1^ significantly increased the frequency of cellular abnormalities in the root meristems of *A. cepa* (Figure 3c), resulting in total CAI values of 9.6% and 11.9%, respectively (Table 1). At 100 ng·L^−1^, chromosomal disorganization during prophase and C-metaphase was observed (Table 1; Figure 4C,D). At 1000 ng·L^−1^, these alterations were also observed, together with sticky chromosomes at metaphase, an alteration not detected at the lower ASA concentration (Table 1; Figure 4C–E). The occurrence of C-metaphases and chromosomal disorganization during prophase is consistent with aneugenic effects, suggesting interference with chromosome organization and/or segregation during mitosis.

### 3.3. Oxidative Stress in Roots

**Figure 5:**
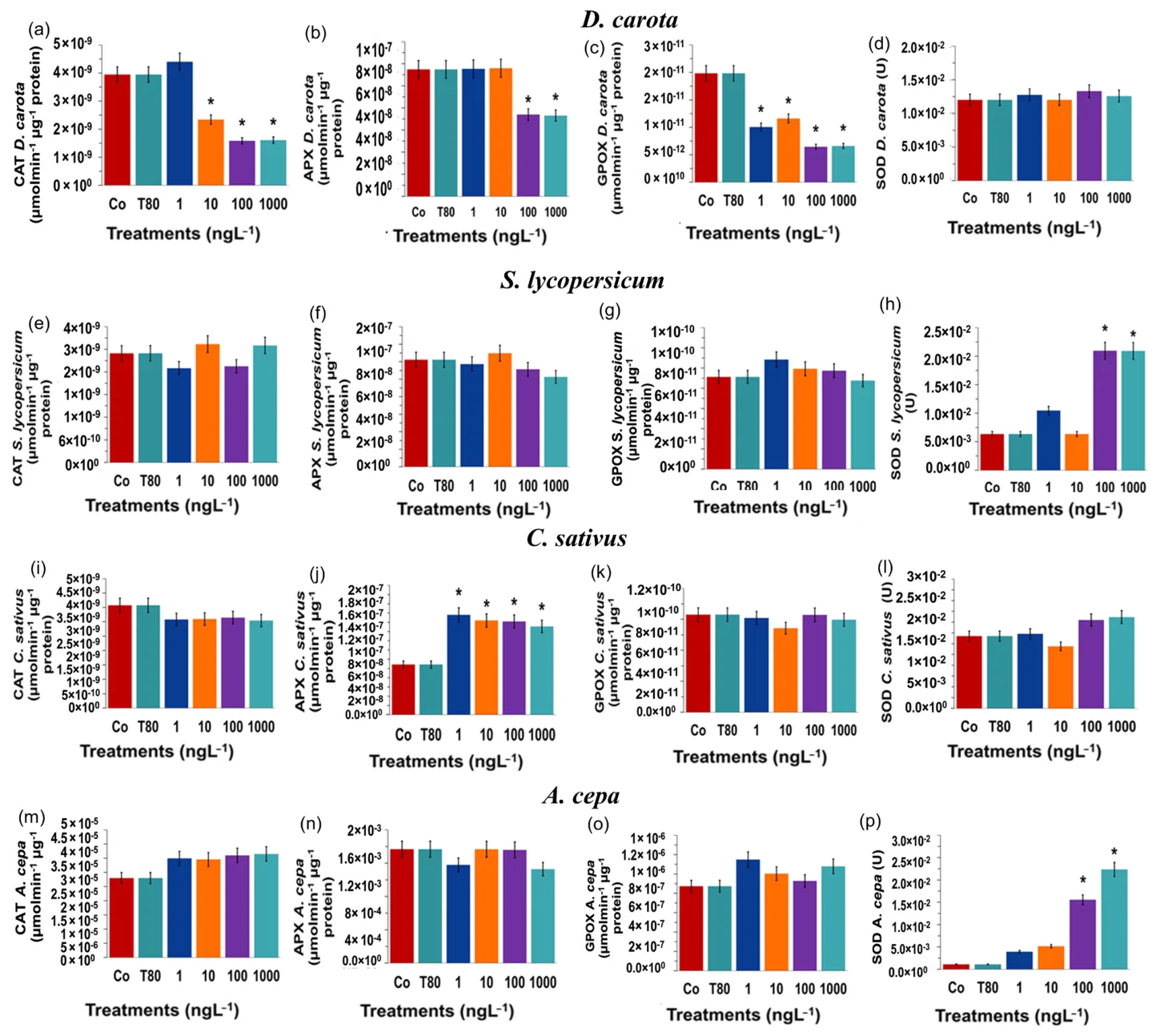
Activity of the enzymes catalase (CAT), ascorbate peroxidase (APX), guaiacol peroxidase (GPOX), and superoxide dismutase (SOD) in *Daucus carota* L., *Solanum lycopersicum* L., *Cucumis sativus* L., and *Allium cepa* L. roots exposed to acetylsalicylic acid, in concentrations of 1, 10, 100, and 1000 ng·L^−1^. * Significant differences from the distilled water control according to Kruskal–Wallis H, followed by Dunn’s post hoc test (*p* ≤ 0.05). Co—Distilled water control. T80—Tween 80 at a concentration of 1000 ng·L^−1^. (**a**) CAT in *D. carota*; (**b**) APX in *D. carota*; (**c**) GPOX in *D. carota*; (**d**) SOD in *D. carota*; (**e**) CAT in *L. esculentum*; (**f**) APX in *L. esculentum*; (**g**) GPOX in *L. esculentum*; (**h**) SOD in *L. esculentum*; (**i**) CAT in *C. sativus*; (**j**) APX in C. sativus; (**k**) GPOX in *C. sativus*; (**l**) SOD in *C. sativus*; (**m**) CAT in *A. cepa*; (**n**) APX in *A. cepa*; (**o**) GPOX in *A. cepa*; (**p**) SOD in *A. cepa*.

In roots, the antioxidant enzymes CAT, APX, GPOX, and SOD are essential for maintaining redox homeostasis and for regulating cell cycle progression in meristems, as they control intracellular levels of reactive oxygen species (ROS) during cell proliferation and differentiation [54,55,56]. In *D. carota*, CAT activity showed a significant reduction starting at 10 ng·L^−1^, remaining inhibited at concentrations of 100 and 1000 ng·L^−1^ (Figure 5a), while APX showed a significant reduction at the two highest concentrations (Figure 5b). In turn, GPOX activity showed a significant reduction as early as 1 ng·L^−1^ (Figure 5c), indicating a pronounced response of this enzyme to ASA exposure. The inhibition of CAT, combined with reduced GPOX and APX activity, suggests a reduced capacity for H_2_O_2_ detoxification, from which an increase in intracellular H_2_O_2_ levels can be inferred, consistent with disruption of cellular redox homeostasis under ASA exposure.

In *S. lycopersicum* and *A. cepa*, SOD activity showed a significant increase at concentrations of 100 and 1000 ng·L^−1^ (Figure 5h,p). In contrast, CAT, APX, and GPOX activities remained unchanged at all concentrations evaluated (Figure 5e–g,i,k,m–o). This enzymatic pattern indicates increased superoxide anion (O_2_•^−^) dismutation, with consequent H_2_O_2_ formation, while the absence of a corresponding CAT and APX response suggests a limited capacity for H_2_O_2_ detoxification. Thus, an increase in intracellular H_2_O_2_ levels can be inferred from the coordinated modulation of the antioxidant enzymes, consistent with disruption of cellular redox homeostasis. In *C. sativus*, no significant changes were observed in CAT, GPOX, and SOD activities (Figure 5i,k,l). However, the significant increase in APX activity across all concentrations (Figure 5j) indicates activation of the antioxidant system in response to alterations in cellular redox balance. The increase in APX activity, in the absence of changes in CAT, GPOX, and SOD, suggests an increased demand for H_2_O_2_ detoxification and is consistent with an increase in H_2_O_2_ levels in the roots.

**Figure 6:**
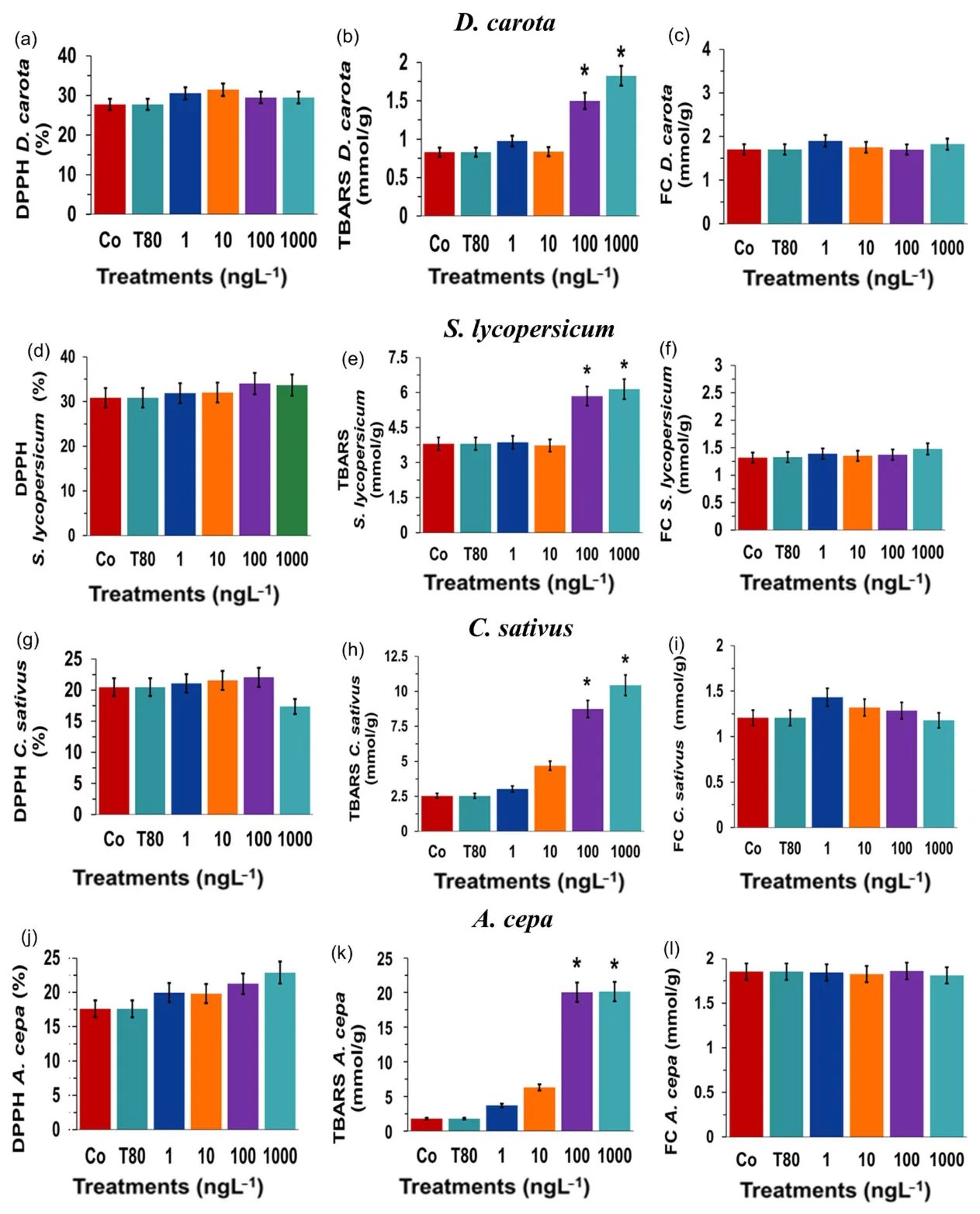
Antioxidant activity (DPPH), total phenolic content (TP), and lipid peroxidation (TBARs) in the roots of *Daucus carota* L., *Solanum lycopersicum* L., *Cucumis sativus* L., and *Allium cepa* L. exposed to acetylsalicylic acid, at concentrations of 1, 10, 100, and 1000 ng·L^−1^. * Significant difference from the distilled water control according to Kruskal–Wallis H, followed by Dunn’s post hoc test (*p* ≤ 0.05). Co—Distilled water control. T80—Tween 80 at a concentration of 1000 ng·L^−1^. (**a**) DPPH in *D. carota*; (**b**) TBARS in *D. carota*; (**c**) FC in *D. carota*; (**d**) DPPH in *L. esculentum*; (**e**) TBARS in *L. esculentum*; (**f**) FC in *L. esculentum*; (**g**) DPPH in *C. sativus*; (**h**) TBARS in *C. sativus*; (**i**) FC in *C. sativus*; (**j**) DPPH in *A. cepa*; (**k**) TBARS in *A. cepa*; (**l**) FC in *A. cepa*.

Based on non-enzymatic biochemical parameters, no significant changes were observed in total antioxidant activity or phenolic compound content in the roots of *D. carota*, *S. lycopersicum*, *C. sativus*, and *A. cepa* at any of the evaluated ASA concentrations (Figure 6a,c,d,f,g,i,j,l). The absence of significant changes in these parameters suggests that the non-enzymatic antioxidant system did not mount a measurable compensatory response to ASA exposure, particularly at concentrations of 100 and 1000 ng·L^−1^. However, at these concentrations, lipid peroxidation was observed in the four evaluated species (Figure 6b,e,h,k), providing evidence of membrane lipid damage and supporting the occurrence of oxidative damage under ASA exposure.

The occurrence of lipid peroxidation (Figure 6b,e,h,k), even in the absence of consistent GPOX modulation (Figure 5c,g,k,o) in *D. carota*, *S. lycopersicum*, *C. sativus*, and *A. cepa*, indicates that, under the experimental conditions evaluated, oxidative damage to cell membranes did not depend exclusively on the activity of this enzyme, but rather on a broader imbalance between the production and detoxification of ROS, particularly involving H_2_O_2_ homeostasis (Figure 5). Considering that intracellular control of H_2_O_2_ depends largely on the coordinated action of CAT and APX, the absence of a compensatory non-enzymatic antioxidant response (Figure 6a,c,d,f,g,i,j,l), coupled with the observed modulation of the enzymatic antioxidant system, is consistent with a resulting redox imbalance and oxidative damage to cell membranes.

Therefore, based on the modulation of CAT, APX, SOD, and GPOX (Figure 5), ASA caused species-dependent alterations in the enzymatic antioxidant system, which were more pronounced at concentrations of 100 and 1000 ng·L^−1^, with the most pronounced response in *D. carota*, an intermediate response in *S. lycopersicum* and *A. cepa*, and the least pronounced response in *C. sativus*.

### 3.4. Dose-Dependent Biological Responses to ASA: Redox, Cytogenetic, and Phytotoxic Effects

The biochemical results (Figure 5 and Figure 6) suggest that ASA exposure affected redox balance in the plant roots and may be related to the cytotoxic, genotoxic, and phytotoxic responses observed. Changes in antioxidant enzyme activities were detected in *D. carota*, *S. lycopersicum*, *C. sativus*, and *A. cepa* (Figure 5), together with increased lipid peroxidation (Figure 6). Since ROS levels were not directly quantified in the present study, these results provide evidence of changes in cellular redox balance and oxidative damage rather than direct evidence of increased ROS production. Lipid peroxidation can generate reactive products, such as lipid hydroperoxides and reactive carbonyl compounds, which may contribute to cellular damage [57,58].

Changes in redox balance may also affect cell-cycle regulation and the organization of the mitotic apparatus [57,59]. Oxidative damage can interfere with DNA and proteins involved in cell division, while changes in microtubule dynamics may affect chromosome organization and segregation [59,60,61]. Reactive products generated during lipid peroxidation may also contribute to oxidative modifications of proteins and chromatin-associated components, potentially affecting chromatin structure and cellular regulation [57,62]. In the present study, altered antioxidant activity and lipid peroxidation were accompanied by reduced mitotic activity, chromosomal abnormalities, and root growth inhibition (Figure 2, Figure 3, Figure 4, Figure 5 and Figure 6). Together, these responses support the involvement of altered redox balance in the cellular and developmental effects observed after ASA exposure.

The reduction in root growth at 100 and 1000 ng·L^−1^ was accompanied by decreased mitotic activity and chromosomal abnormalities in *A. cepa* (Figure 2, Figure 3 and Figure 4). Because cell proliferation in the root meristem is closely associated with root elongation and tissue formation [63], the cytotoxic and genotoxic responses may have contributed to the inhibition of root growth. The occurrence of C-metaphases, prophase disorganization, and sticky chromosomes further indicates interference with normal mitotic progression [64]. These results show that the effects of ASA were not restricted to biochemical responses but also extended to cellular processes associated with root development.

ASA may undergo partial hydrolysis to salicylic acid under biological conditions [64,65]. Salicylic acid is involved in several physiological and stress-related processes in plants and can influence antioxidant responses and redox regulation [65,66]. In the present study, salicylic acid was detected during ASA exposure, but only at low levels (Figure 1), indicating limited conversion under the experimental conditions. Thus, the contribution of salicylic acid to the responses observed was likely limited under the conditions evaluated.

The responses observed at different ASA concentrations do not indicate a typical hormetic pattern. Hormesis generally involves stimulation at low concentrations followed by inhibition at higher concentrations [66,67]. In the present study, the lowest concentrations did not produce significant stimulatory effects, whereas phytotoxic, cytotoxic, and genotoxic responses were observed at 100 and 1000 ng·L^−1^. Therefore, the present findings are more consistent with a concentration-dependent response than with a classical hormetic pattern.

ASA and salicylic acid have also been investigated as exogenous treatments in plants under agronomic conditions. Senaratna et al. [17] reported increased stress tolerance in bean and tomato plants following exposure to ASA at 0.1–0.5 mM (approximately 18–90 mg L^−1^). In that study, ASA was also applied to 14-day-old seedlings by soil drenching. ASA has also been evaluated under field conditions in potato cultivation at concentrations of 0.2, 0.4, 0.6, and 0.8 mM (approximately 36, 72, 108, and 144 mg L^−1^) [68,69]. The first application was performed by immersing the seed tubers, followed by four foliar applications at two-week intervals over 105 days. The 0.4 mM treatment (72 mg L^−1^) favored sprouting, vegetative vigor, and flowering, although no significant increases in potato yield or biomass were observed [68,70]. These agronomic studies demonstrate that ASA can modulate plant physiological and developmental responses when supplied at mg L^−1^ concentrations. In contrast, the present study investigated trace concentrations (1–1000 ng·L^−1^) relevant to environmental exposure and identified biochemical, cytotoxic, genotoxic, and root-growth responses within this concentration range. Thus, the cytogenetic and phytotoxic effects observed in the present study occurred at concentrations several orders of magnitude lower than those associated with the agronomic responses reported in previous studies. The biological response to ASA may therefore vary with dose, with concentrations used in agronomic applications producing physiological modulation, whereas much lower concentrations may affect cellular processes such as cell division and chromosome integrity. The contrasting responses may therefore reflect differences in concentration, exposure context, and biological endpoints, rather than an inconsistency in the effects of ASA.

The developmental stage and tissue type may also influence the response to ASA. Plant metabolism is spatially and developmentally regulated, and different cell types and tissues have distinct metabolic capacities and physiological functions [70,71]. This distinction is particularly relevant when comparing actively dividing meristematic tissues with more differentiated tissues. The root apical meristem is characterized by intense cell proliferation and a highly dynamic cellular state, making disturbances affecting cell division particularly relevant to root development [72,73]. In contrast, differentiated tissues have distinct metabolic and physiological characteristics that may influence compound uptake, transformation, detoxification, and cellular responses [73]. It is therefore possible that ASA may produce less pronounced effects in differentiated tissues than in actively dividing root meristems, although this hypothesis requires experimental confirmation.

From an environmental perspective, the cytogenetic effects observed at 100 and 1000 ng·L^−1^ indicate that ASA can affect plant cells even at trace concentrations. Importantly, these concentrations are several orders of magnitude lower than those commonly used in agronomic applications [17,68] and fall within the trace concentration range relevant to environmental exposure. Nevertheless, the occurrence and bioavailability of ASA in agricultural soils may be influenced by soil properties, sorption, transformation, degradation, and other processes. Thus, the present findings provide relevant evidence of the potential effects of trace ASA concentrations on plant cellular processes, while field-based studies under realistic soil and environmental conditions will be important to determine whether these effects are also expressed under natural exposure conditions.

The use of multiple plant models and complementary biomarkers strengthens the assessment of the biological effects of ASA at trace concentrations. The responses observed in *D. carota*, *S. lycopersicum*, *C. sativus*, and *A. cepa* encompassed biochemical, cellular, genotoxic, and developmental endpoints, providing evidence of effects across different levels of biological organization. In particular, *A. cepa* was included as a cytogenetic model because its actively dividing root meristem enables the detection of alterations in mitotic activity and specific chromosomal abnormalities [26,32]. In the present study, this model provided direct evidence of cytogenetic effects induced by ASA, complementing the biochemical and growth-related responses observed in the other plant models. The consistency among these responses indicates that the effects were not restricted to a single biomarker or level of biological organization. Nevertheless, species-specific differences in uptake, metabolism, antioxidant capacity, cell-cycle regulation, and sensitivity should be considered when extending these findings to other plant species and organisms. Overall, the integration of multiple plant models and complementary endpoints provides a robust assessment of plant responses to trace ASA concentrations under controlled conditions.

These findings contribute to advancing the understanding of the biological activity of ASA at environmentally relevant concentrations and provide a basis for further investigation under more complex and environmentally realistic conditions, including soil–plant systems, to determine how these responses translate to natural environments.

## 4. Conclusions

The results support the hypothesis that exposure to trace concentrations of ASA can impair root development and induce cytogenetic alterations and modulate biochemical responses associated with redox homeostasis. The most pronounced effects were observed at 100 and 1000 ng·L^−1^, with cytogenetic alterations detected in *A. cepa* and more pronounced at 1000 ng·L^−1^.

Under the conditions evaluated, ASA exposure induced phytotoxic, cytogenetic, and biochemical responses across the plant models investigated, whereas seed germination remained unaffected. The occurrence of responses at different levels of biological organization, including root growth, antioxidant responses, lipid peroxidation, and chromosomal alterations, provides experimental evidence that trace concentrations of ASA can affect plant biological processes.

These results expand the current understanding of the biological activity of ASA at environmentally relevant concentrations and support the need for further investigation under more complex and environmentally realistic conditions, particularly in soil/plant systems, to determine the occurrence and ecological significance of these responses in natural environments.

## Figures and Tables

**Table 1:** Number and types of cellular alterations and Cellular Alteration Index observed in root meristems of *Allium cepa* L. bulbs exposed to acetylsalicylic acid at concentrations of 1, 10, 100, and 1000 ng·L^−1^.

	Total Cellular Changes				
Treatment	Micronucleus	Chromosomal Disorganization in Prophase	Metaphase C	Sticky Chromosomes at Metaphase	CAI (%) ± SD
Co	03	00	07	00	0.5 ± 0.3
T80	01	00	09	00	0.5 ± 0.3
1 ng·L^−1^	00	10	06	00	0.8 ± 0.5
10 ng·L^−1^	00	17	03	00	1.0 ± 0.5
100 ng·L^−1^	00	110	82	00	9.6 ± 1.5 *
1000 ng·L^−1^	00	64	59	115	11.9 ± 1.5 *

* Significant difference from the distilled water control according to the Kruskal–Wallis H test followed by Dunn’s post hoc test (*p* ≤ 0.05). Co—distilled water control; T80—Tween 80 at a concentration of 1000 ng·L^−1^; CAI—Cellular Alteration Index; SD—standard deviation.

## Data Availability

The original contributions presented in this study are included in the article. Further inquiries can be directed to the corresponding authors.

## Abbreviations

The following abbreviations are used in this manuscript:

ASA	Acetylsalicylic Acid
ROS	Reactive Oxygen Species
BOD	Biochemical Oxygen Demand
RGI	Relative Growth Index
GI	Germination Index
ARL	Average Root Length
MI	Mitotic Index
CAI	Cellular Alteration Index
CAT	Catalase
APX	Ascorbate peroxidase
GPX	Guaiacol peroxidase
SOD	Superoxide dismutase
U	Enzyme unit
FC	Folin–Ciocalteu
